# Effect of emulsification methods on the physicochemical properties of emulsion stabilized by calcium carbonate and sodium alginate

**DOI:** 10.3389/fnut.2022.977458

**Published:** 2022-09-02

**Authors:** Xiaotong Yang, Haomin Sui, Hongshan Liang, Bin Li, Xiangxing Yan, Jing Li

**Affiliations:** ^1^College of Food Science and Technology, Huazhong Agricultural University, Wuhan, China; ^2^School of Transportation, Wuhan University of Technology, Wuhan, China

**Keywords:** calcium carbonate, sodium alginate, preparation method, co-stabilized, Pickering emulsion

## Abstract

Our lab’s studies have found that heavy calcium carbonate (CaCO_3_) with sodium alginate (SA) can synergistically stabilize Pickering emulsion. However, there were significant differences in the flow characteristics of the emulsions obtained by different preparation methods during storage. Herein, in this current work, Pickering emulsions were prepared by two-step emulsifying method (SA was added into the primary emulsion stabilized by CaCO_3_ for secondary shearing, M1) and one-step emulsifying method (oil phase was added to homogeneous dispersed CaCO_3_-SA solution for one-step shearing, M2), respectively. The particle size, microstructure, rheology and microrheological properties of these two kinds of emulsions and the interaction of CaCO_3_ with SA were analyzed. The results showed that the droplet size of M1 emulsion was 21.78–49.62 μm, and that of M2 emulsion was 6.50–11.87 μm. M1 emulsion had stronger viscoelasticity, and could transform into a gel state during storage. However, M2 emulsion remained in flow condition all the time which was related to the interaction between SA and CaCO_3_ in the aqueous phase.

## Introduction

Because of its unique stability, Pickering emulsion has been considered promising in the food, medicine, cosmetics industries and the like, which enable the efficiently carrying and selectively delivering of active ingredients (1–3). Nano or micron sized solid particles including inorganic particles (4–6), protein particles (7–9), polysaccharide particles (10–12), protein-polysaccharide complexes (13–15), and lipid crystal particles (16, 17) were used as stabilizers instead of surfactants to stabilize Pickering emulsion (18). Food grade calcium carbonate (CaCO_3_) is a kind of inorganic particle approved for use in food. It has the characteristics of wide source, easy access, low price and convenient production. Importantly, it can be used as stabilizer for food grade Pickering emulsion. However, CaCO_3_ particles with small particle size have strong interface energy and are always easy to agglomeration, which is difficult to form stable emulsion (19). Therefore, the surface of CaCO_3_ particles is usually modified to improve its hydrophilicity/hydrophobicity, such as grafting active groups onto the surface of particles (20). Meanwhile, the stability of emulsion can be improved to a certain extent by introducing food-grade polymer stabilizers such as protein and polysaccharides. Sodium alginate (SA), as a natural polysaccharide, is often used as thickener, gelling agent, preservative, or emulsifier in food industry (21). Studies have shown that SA can effectively promote the interfacial adsorption and enhance the emulsifying properties of inorganic particles (22–24). Our laboratory’s preliminary research also found that SA can synergistically stabilize Pickering emulsion with CaCO_3_. However, there were significant differences in the flow characteristics of the emulsions obtained by different preparation methods during storage.

Herein, in this paper, Pickering emulsion M1 and M2 stabilized by CaCO_3_ and SA were prepared by two-step emulsifying method and one-step emulsifying method, respectively. Thereinto, the two-step emulsifying method was to prepare the primary emulsion stabilized by CaCO_3_ first, and then added SA solution for secondary shearing to obtain M1 emulsion. The one-step emulsifying method was to first disperse CaCO_3_ in SA solution, and then oil phase was added to obtain M2 emulsion through one-time shearing. The effect of different preparation methods on the physicochemical properties and stability of emulsion was explored by measuring the particle size, microstructure, rheology and microrheological properties of two kinds of emulsions.

## Materials and methods

### Materials

Soybean oil was purchased from Yihai Kerry Co., Ltd. (Shenzhen, China). Heavy CaCO_3_ was purchased from Zhengzhou Ruipu Bioengineering Co., Ltd. (Henan, China). SA was purchased from Qingdao Haizhilin Biological Co., Ltd. (Shandong, China).

### Preparation of emulsion

#### Preparation of calcium carbonate particles

Before ball milling, food grade heavy CaCO_3_ was dried in an oven at 60°C for 24 h, and then milled by high-energy nano ball mill (CJM-SY-B, Qinhuangdao Taiji Ring Nano-Products Co., Ltd., Hebei, China). After 8 h, the sample was taken out to obtain CaCO_3_ particles with smaller particle size, which were named CaCO_3_-8.

#### Preparation of emulsion by two-step emulsifying method

Accurately weighed SA powder (1.5, 2, 2.5, and 3 g) were dissolved in ultrapure water (50 g). It was dispersed for 2 h at room temperature with an electric mixer (HD2010W, Shanghai Sile Instrument Co., Ltd., Shanghai, China) at 800 r/min to obtain uniform SA solution, and then placed in refrigerator at 4°C overnight. Accurately weighed CaCO_3_-8 (1.7 g) was dispersed in ultrapure water (15 g) with 8.5 wt% CaCO_3_. Then soybean oil (4 g) was added to the aqueous phase, making the water-to-oil ratio 5:1. A high-speed shearing machine (Ultra-Turrax T25, IKA Works Guangzhou, Guangzhou, China) was used to shear it at 12,000 r/min for 1 min to obtain the Pickering initial emulsion stabilized by CaCO_3_. Subsequently, SA solutions (5.15, 5.2, 5.25, and 5.3 g) were added to the initial emulsion, so that the final concentration of SA in the emulsion system was 0.75, 1, 1.25, and 1.5 wt% of the aqueous phase. Finally, the final emulsion was obtained by shearing with a high-speed shearing machine at 12,000 r/min for 5 min. The Pickering emulsion prepared by this method was named M1. The prepared emulsion was stored at room temperature (25°C) and added 0.02% sodium azide to inhibit the growth of microorganisms and facilitate subsequent measurement (25).

#### Preparation of emulsion by one-step emulsifying method

Accurately weighed SA powder (0.15, 0.2, 0.25, and 0.3 g) were dissolved in ultrapure water (20 g). A magnetic stirrer (SP-300, Hangzhou Miu Instrument Co., Ltd., Zhejiang, China) was used to disperse it at 550 r/min for 1.5 h to prepare SA solution with concentration of 0.75, 1, 1.25, and 1.5 wt%, stored at 4°C overnight. Accurately weighed CaCO_3_-8 (1.7 g) was dispersed in SA solutions with different concentrations. A magnetic stirrer was used to disperse it at 550 r/min for 0.5 h to obtain an aqueous phase with 8.5 wt% CaCO_3_. Then soybean oil (4 g) was added to the aqueous phase as the oil phase to make the water-to-oil ratio 5:1. A high-speed shearing machine was used to shear it at 12,000 r/min for 6 min to obtain the Pickering emulsion stabilized by CaCO_3_ and SA. The Pickering emulsion prepared by this method was named M2. The prepared emulsion was stored at room temperature (25°C) and added 0.02% sodium azide for further measurement.

### Determination of particle size of emulsion

The particle size distribution and the average particle size of the emulsion were measured by laser particle size analyzer (Mastersizer 2000, British Malvern Instrument Co., Ltd., United Kingdom). Water (refractive index: 1.333) was selected as dispersant, soybean oil (refractive index: 1.475) was selected as sample material. The setting range of laser shading index was 1–20%, and the pump speed was 2,000 r/min (26). The result of the emulsion particle size was expressed by the volume mean diameter (D[4, 3]). The calculation formula was as follows:


$$D\,\left[4,3\right]=\Sigma n_{i}d_{i}^{4}/\Sigma n_{i}d_{i}^{3}$$

Where d_*i*_ was the droplet diameter of emulsion (μm), n_*i*_ was the number of emulsion droplets with a particle size of d_*i*_.

### Determination of creaming index

The degree of emulsification of emulsion was represented by creaming index (CI) (27). The fresh emulsion was stored at room temperature and the height of the supernatant layer and emulsified layer were recorded on the 0th, 1st, 2nd, 7th, 14th, and 28th days after storage. The computational formula of CI of the emulsion was as follows:


$$CI(\%)=H_{s}/H_{t}\times 100$$

Where H_*s*_ indicated the height of the supernatant layer of the emulsion (cm), H_*t*_ indicated the height of the emulsion (cm).

### Observation of micromorphology

#### Optical microscope

The microstructure of emulsion was observed by optical microscope (OD1400Y, Ningbo Sunny Instruments Co., Ltd., Zhejiang, China) under 20 times objective lens, and the ubiquitous droplet structure was selected to take pictures.

#### Cryo-scanning electron microscopes

The samples were quickly transferred to the vacuum preparation room after cryopreservation by liquid nitrogen. The surface of the droplets was cut by cooling knife to form a section. It was sublimated at −95°C for 4 min and sputter coated with platinum (28). The prepared sample was transferred to a field emission scanning electron microscope (SEM) (SU8010, Techcomp Instrument Co., Ltd., Shanghai, China) with a constant temperature of −130°C. The microstructure of the emulsion was observed under the condition of 1.50 and 5.00 k times.

### Determination of rheological properties

The rheological properties of the emulsion at different storage time were measured on the 0th, 14th, and 28th days by rheometer (DHR2, TA Instrument Co., Ltd., United States). The aluminum flat plate with a diameter of 60 mm was selected as the experimental measuring fixture, and the measuring gap was set as 500 μm. The dynamic frequency sweep measurement was carried out in the frequency range of 0.1–100.0 rad/s with 0.06% strain. The shear rate of the flow sweep was 0.01–100.0 s^–1^, and the frequency was fixed at 1 Hz. The measurement temperature was constant at 25°C (29).

### Determination of microrheological properties

The microrheological properties of the emulsion were measured and analyzed by an optical micro-rheometer (Rheolaser Master, Formulaction Instrument Co., Ltd., France) on the 0th, 14th, 28th, 42nd, and 56th days. The measurement temperature was set to a constant temperature of 25°C, and the sample information was obtained by scanning every 150 s. The solid-liquid equilibrium balance (SLB) values, elasticity index (EI), and macroscopic viscosity index (MVI) were calculated by RheoSoft Master software through the mean square displacement (MSD) curve (30).

### Interaction of sodium alginate with calcium carbonate

#### Stability analysis of turbiscan

The aggregation and dispersion stability of CaCO_3_/SA suspension was measured by a multiple-light Scattering instrument (Turbiscan Tower, French Formulaction Co., Ltd., France). According to the method described by Wang et al. (31) with some modifications. Backscattering was used for sample analysis, scanned every 150 s from 0 to 30 min and every 30 min from 0.5 to 10 h at 25°C. Turbiscan stability index (TSI) was calculated by Towersoft-1.4.0.4 to reflect the overall stability of suspension.

#### Observation on particle morphology of calcium carbonate

The microscopic morphology of CaCO_3_ particles containing and without SA was observed by transmission electron microscope (H-7650, Hitachi Limited, Japan). The newly prepared suspension was diluted 500 times with pure water and then attached to a copper net for drying. After that, the microstructure of CaCO_3_/SA dispersion was observed by transmission electron microscope.

#### Determination and analysis of zeta potential

Zeta potential of CaCO_3_/SA suspension was measured by nano-particle size and zeta potential analyzer (Nano ZS, British Malvern Instrument Co., Ltd., United Kingdom). Water (refractive index 1.333) was selected as dispersant, CaCO_3_ (refractive index 1.658) was selected as the sample material. The temperature was measured at 25°C.

### Data analysis

Each set of experiments was repeated at least three times, and each measurement was set to at least three parallels. The measurement result was the mean ± standard deviation after three measurements. The drawing software used in this paper is Origin 9.

## Results and discussion

### Particle size of calcium carbonate and emulsion droplets

**FIGURE 1:**
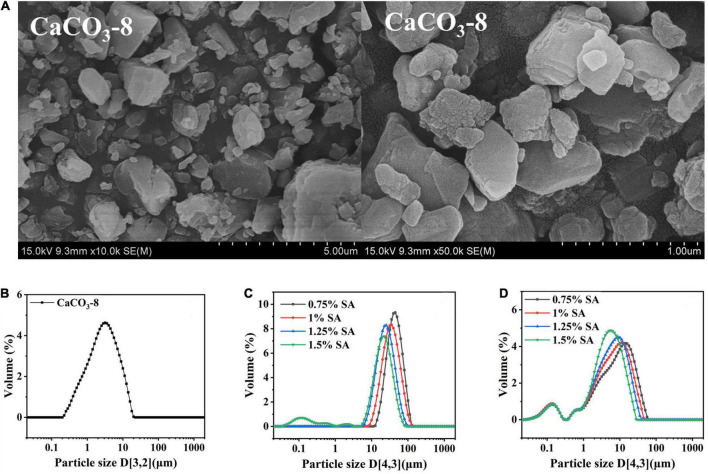
Morphology of CaCO_3_ after 8 h of ball milling: **(A)** image of scanning electron microscope; **(B)** image of particle size distribution; particle size distribution of M1 emulsion **(C)** and M2 emulsion **(D)**.

Seen from the image of TEM, the particle shape was similar to the irregular cube structure, and more particles were presented as loose aggregates (Figure 1A). The particle size of CaCO_3_-8 was 1.883 ± 0.09 μm (Figure 1B).

M1 (two-step emulsifying method) and M2 emulsions (one-step emulsifying method) were prepared by CaCO_3_-8 and SA. The particle size of emulsion droplets is a critical parameter, which has great influence on the characteristics, stability and application. Figures 1C,D were the particle size distributions of freshly prepared M1 and M2 emulsions. With the increase of SA concentration, the particle size of emulsion decreased. It demonstrated that the addition of SA could enhance the stability of emulsion. Compared with Figures 1C,D, different preparation methods had great influence on the particle size. The reason was that the average droplet diameter of the final emulsion depends on the initial addition of particles and the their arrangement on the oil-water interface (32). In the preparation process of M1 emulsion, the Pickering emulsion stabilized by CaCO_3_ was first completed. CaCO_3_ adsorbed on the oil-water interface and formed a strong adsorption layer. Then SA was added, when at low SA concentration, the contact between SA and CaCO_3_ was weak, which lead to weak synergistic stabilizing effect. After the shear was stopped, the droplets were aggregated to reduce the area of the oil-water interface and could not effectively stabilize the emulsion. However, the dispersion and dissolution of SA and CaCO_3_ had been achieved before the preparation of M2 emulsion. The full contact of SA and CaCO_3_ was produced a synergistic emulsification effect, so the emulsification performance was improved.

### Stability of emulsion

**FIGURE 2:**
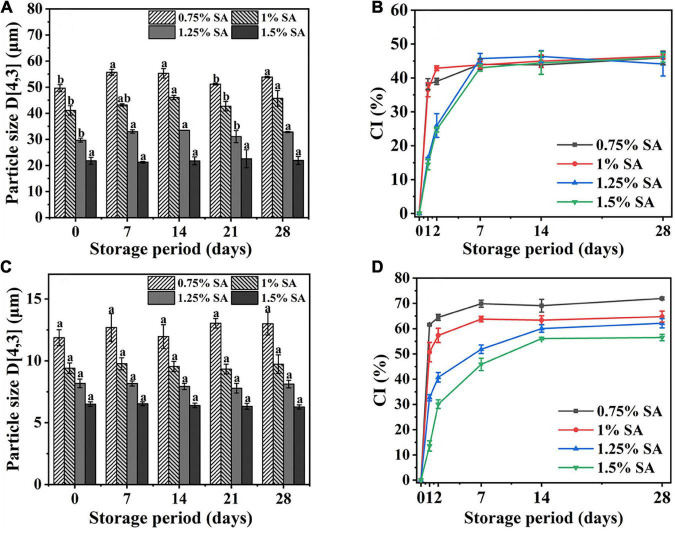
Particle size and CI of M1 emulsion **(A,B)** and M2 emulsion **(C,D)** at different storage times. The measurement results were the average values of triplicate measurements. Different lowercase letters indicated that the particle size of same samples was significantly different during different storage periods (*p* < 0.05).

Figures 2A,C, respectively showed the change of particle size of M1 emulsion and M2 emulsion during the 28-day storage process. The particle size of M2 emulsion had no significant change during storage, while the particle size of M1 emulsion changed slightly. In addition, the particle size of the emulsion changed more significantly with the concentration of SA decreased.

Figures 2B,D, respectively showed the CI changes of M1 emulsion and M2 emulsion during 28-day storage. The CI of M1 and M2 emulsions increased rapidly from 0 to 2 days and reached equilibrium at about 7–14 days. There was no significant difference in CI of M1 emulsion with different SA concentration at 28 days, while CI of M2 emulsion decreased with the increase of SA concentration during 28-day storage. Figure 2B showed that the addition of SA in M1 emulsion only had an impact on the rate of change in CI, which might be because the emulsification of CaCO_3_ played a vital role in M1 emulsion, while the co-emulsification of CaCO_3_ and SA occurred in M2 emulsion. Moreover, there was no oil phase separation in all the emulsions during 28-day storage.

### Micromorphology of emulsion

**FIGURE 3:**
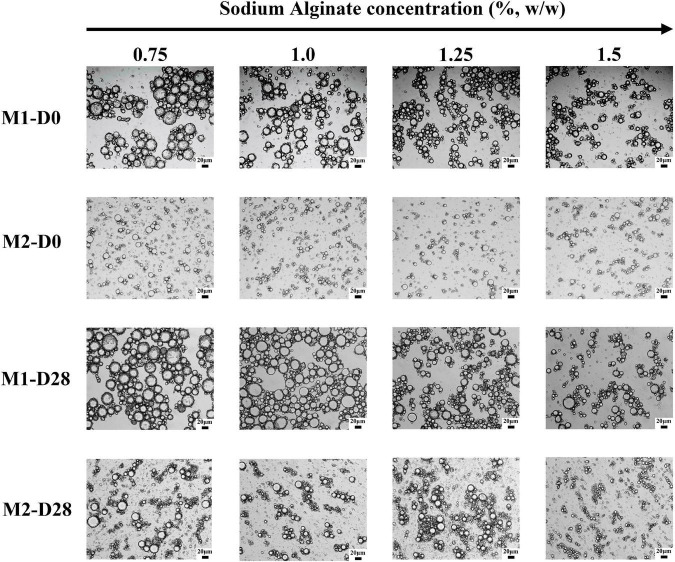
Microstructure images of M1 and M2 emulsions at different storage times. D0 and D28 represent emulsions stored for 0 and 28 days.

Figure 3 was an optical microscope image of emulsion under different storage time. The droplet size of M1 emulsion was larger than that of M2 emulsion. Under the same preparation method, the droplet size of emulsion was reduced effectively with the increase of the concentration of SA. It could also be seen that M1 emulsion had obvious droplet aggregation, which may be related to that the SA added in the second emulsification process was not fully contacted with the CaCO_3_ on the surface of the emulsion droplets. Therefore, SA molecule would interact with multiple emulsion droplets at the same time, resulting in bridging flocculation. In addition, it was found that M1 emulsion and M2 emulsion kept their original microstructure throughout storage.

**FIGURE 4:**
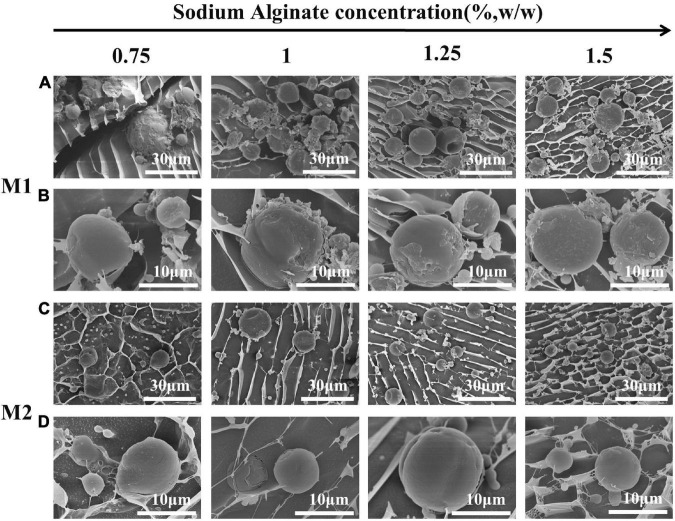
Cryo-SEM images of M1 and M2 emulsions: **(A,C)** the images ×1.50 k; **(B,D)** the images ×5.00 k.

Figure 4 showed the droplet morphology of M1 emulsion and M2 emulsion observed under cryo-scanning electron microscope (cryo-SEM). The surface of M1 emulsion droplets was rough, and there was a three-dimensional network around the droplets. It was confirmed that CaCO_3_ and SA had synergistic effect of stabilizing the emulsion. It also verified the speculation that SA failed to form full contact with CaCO_3_ on the surface of emulsion droplets. M2 emulsion showed a good spherical shape, the surface was smooth and undamaged, and there was also a robust three-dimensional network around the droplets. The results showed that the process of CaCO_3_ and SA dispersion in M2 emulsion achieved the full contact between SA and CaCO_3_ particles, so that both of them could play a synergistic stabilizing role effectively.

### Rheological properties of emulsion

In order to further analyze the causes of emulsion gelation, this paper studied the rheological and microrheological properties of emulsion system during storage, and explored the effect of emulsion preparation methods on emulsion stability.

#### Dynamic frequency sweep

**FIGURE 5:**
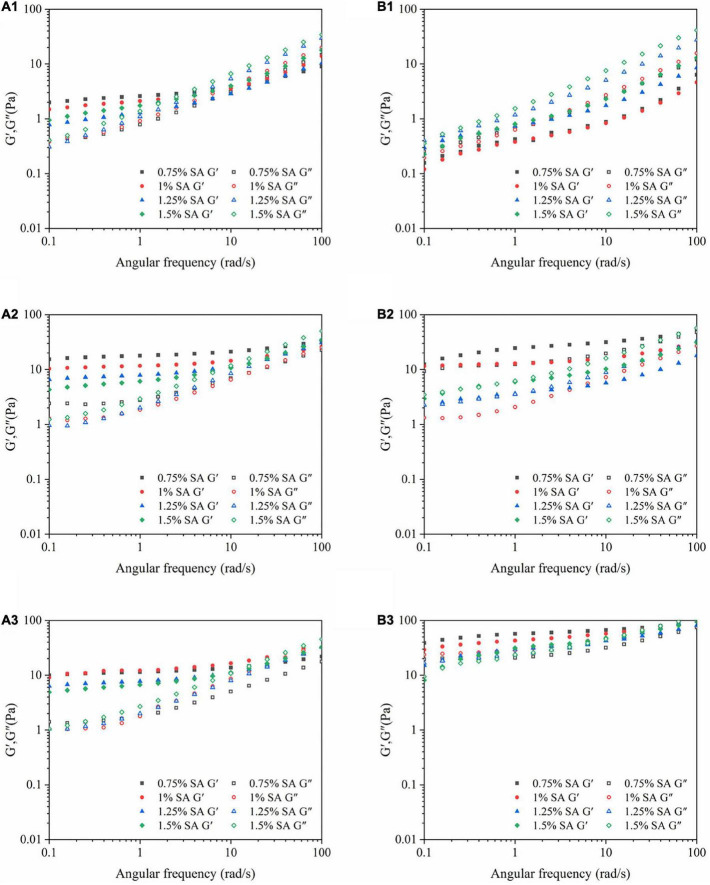
Dynamic frequency sweep images: M1 emulsions stored for 0 **(A1)**, 14 **(A2)**, and 28 **(A3)** days; M2 emulsions stored for 0 **(B1)**, 14 **(B2)**, and 28 **(B3)** days.

Figure 5 showed the results of dynamic frequency sweep of emulsion at different storage time on the 0th, 14th, and 28th days at 25°C. The elastic modulus (G′) and viscous modulus (G″) of the two emulsions increased with the increase of the angular frequency, hinting that the three-dimensional network structure existed in the emulsion. It was speculated that the reason was that both the adsorbed and excessive unabsorbed CaCO_3_ particles on the oil-water interface were electrostatically adsorbed with SA to enhance the gel network structure of the emulsion. Moreover, G″ was greater than G″ in the small frequency range before the intersection, the elastic behavior of the emulsion system was dominant. However, the three-dimensional network structure of emulsion was relatively weak, and it was easy to be destroyed with the increase of angular frequency. With the extension of storage time, the values of G′ and G″ were greatly increased, and the intersection of G′ and G″ was constantly delayed. It revealed that the three-dimensional network structure in the emulsion was continuously strengthened, which was manifested as the enhancement of the viscoelastic of the emulsion. The difference was that G′ and G″ of M1 emulsion intersected in a higher frequency range than M2 emulsion, indicating that M1 emulsion had a strong three-dimensional network structure and gel-like behavior.

#### Flow sweep

**FIGURE 6:**
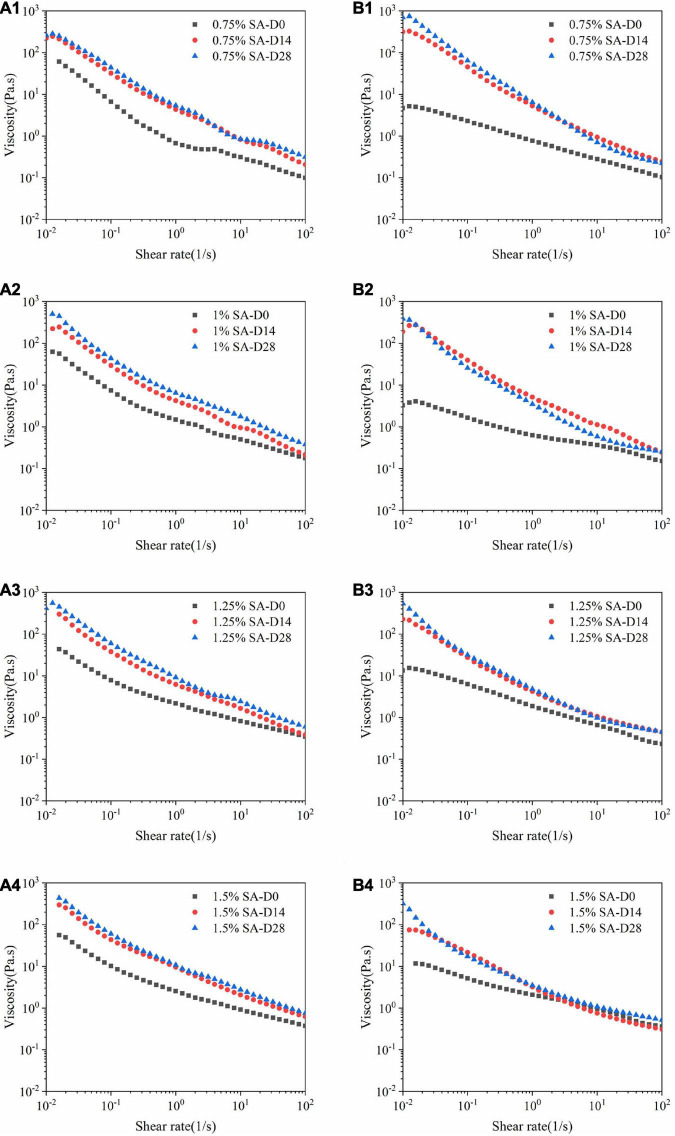
Flow sweep images: M1 emulsions with 0.75% SA **(A1)**, 1% SA **(A2)**, 1.25% SA **(A3)**, and 1.5% SA **(A4)**; M2 emulsions with 0.75% SA **(B1)**, 1% SA **(B2)**, 1.25% SA **(B3)**, and 1.5% SA **(B4)**. D0, D14, and D28 represent emulsions stored for 0, 14, and 28 days.

Figure 6 showed the change in the viscosity of the emulsions with the shear rate at different storage times on the 0th, 14th, and 28th days at 25°C. It can be seen that all emulsions exhibited shear thinning behavior and were non-Newtonian fluids. The results showed that with the extension of storage time, the viscosity of M1 and M2 emulsions increased. This may be because some of calcium ions dissociated from CaCO_3_ are continuously released into the system to combine with the G block in the alginate during the storage process, thereby cross-linking to form an “egg box structure” (33). Furthermore, the three-dimensional network structure in M1 and M2 emulsions was continuously strengthened with the extension of storage time. Thus, the restriction on the movement of emulsion droplets increased, which was macroscopically manifested as an increase in viscosity. At the same time, the three-dimensional network structure in M1 and M2 emulsions was gradually destroyed and reduced as the shear rate increases.

### Microrheological properties of emulsion

**FIGURE 7:**
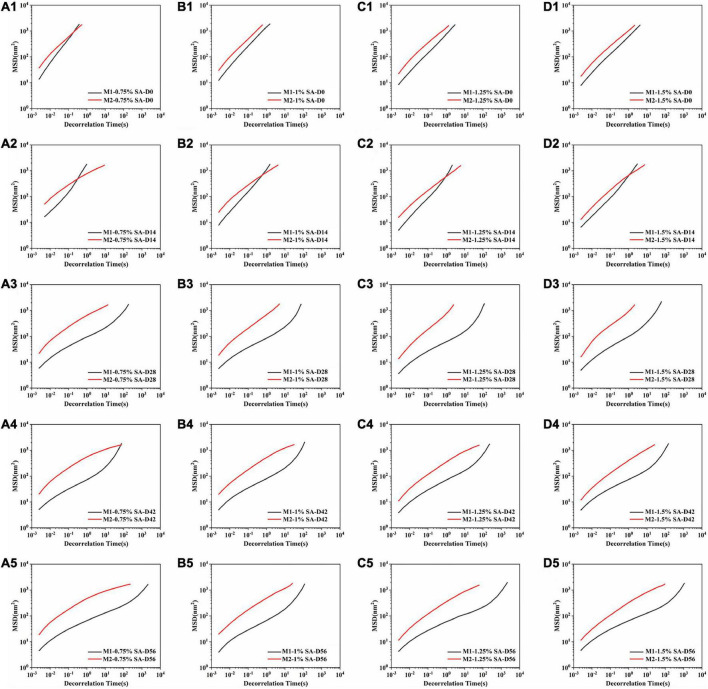
Mean squared displacement curve: M1 and M2 emulsions containing 0.75% SA **(A1–A5)**, 1% SA **(B1–B5)**, 1.25% SA **(C1–C5)**, and 1.5% SA **(D1–D5)** were stored for 0, 14, 28, 42, and 56 days. D0, D14, D28, D42, and D56 represent emulsions stored for 0, 14, 28, 42, and 56 days.

The micro-rheology method explained the microrheological properties of the sample by detecting Brownian motion of scattered particles. Therefore, the viscoelasticity of the emulsion could be measured without mechanical shear, and the structure of the sample would not be damaged. The MSD was obtained by tracking the Brownian motion of the particles in the sample to analyze microrheological properties of the system. Figure 7 showed the MSD curves of emulsion samples at 0, 14, 28, 42, and 56 days at 25°C. MSD curves of M1 and M2 fresh emulsions rose linearly with time, representing that the emulsion particles diffused freely in the solution, and they were pure viscous emulsions. From the 14th day, MSD curves of M1 and M2 emulsions showed different trends. MSD curves of M1 entered the relaxation platform, which indicated that the movement of emulsion particles was restricted, and the particles moved in the “cage” formed by interaction with other particles (34). These interactions made the sample elastic, that was, M1 showed obvious viscoelasticity. In contrast, the change of M2 emulsion was not obvious, namely its elasticity and viscosity were lower than M1 emulsion. In addition, the slope of MSD curve decreased with the increase of storage time, the de-correlation time of MSD curve gradually became longer, and the viscosity of M1 and M2 increased continuously. These results were consistent with the flow sweep results of rheology.

It could be seen from Table 1 that the SLB value of the freshly prepared emulsion was between 0.5 and 1, indicating that the emulsion system was dominated by liquid behavior at this time. The SLB value showed a decreasing trend with the prolongation of storage time, indicating that the emulsion system gradually changed from the liquid property to the elastomer property. After storage for 28 days, the SLB value of M1 emulsion decreased significantly and the SLB value was <0.5, indicating that the movement rate of droplets in M1 emulsion system was limited, and the formation of three-dimensional network structure led to elastic behavior dominating in emulsion. Extending the storage time, the SLB value of M1 emulsion decreased gradually from 0 to 0.5, while the SLB value of M2 emulsion always ranged from 0.5 to 1. It turned out that M1 emulsion was more easily changed into gel state during storage, and M2 could maintain its fluidity for a long time. It was confirmed that the different preparation methods of CaCO_3_/SA emulsion would affect the structure of emulsion during storage.

**TABLE 1:** Solid liquid balance values of M1 emulsion and M2 emulsion of different storage time.

Emulsion	SA concentration (%)	SLB				
		D0	D14	D28	D42	D56
M1	0.75	0.93 ± 0.11	0.73 ± 0.08	0.46 ± 0.01	0.42 ± 0.00	0.42 ± 0.01
	1	0.67 ± 0.02	0.71 ± 0.05	0.45 ± 0.02	0.42 ± 0.01	0.42 ± 0.01
	1.25	0.69 ± 0.02	0.65 ± 0.06	0.46 ± 0.02	0.46 ± 0.00	0.42 ± 0.00
	1.5	0.67 ± 0.01	0.64 ± 0.01	0.49 ± 0.02	0.43 ± 0.01	0.43 ± 0.02
M2	0.75	0.64 ± 0.02	0.52 ± 0.01	0.53 ± 0.01	0.51 ± 0.01	0.52 ± 0.00
	1	0.62 ± 0.04	0.55 ± 0.01	0.56 ± 0.03	0.54 ± 0.00	0.54 ± 0.00
	1.25	0.61 ± 0.01	0.59 ± 0.01	0.61 ± 0.02	0.55 ± 0.01	0.55 ± 0.00
	1.5	0.63 ± 0.01	0.64 ± 0.01	0.61 ± 0.01	0.56 ± 0.00	0.55 ± 0.00

D0, D14, D28, D42, and D56 represent different storage times of emulsions.

SA represents sodium alginate and SLB represents the solid liquid balance values.

As shown in Table 2, the EI value continuously increased with the extension of storage time. It indicated that the elasticity of the emulsion system increased with time, and this phenomenon was consistent with the results of dynamic frequency sweep. Comparing the EI value of M1 and M2 emulsions, the EI value of M1 emulsion was always higher than that of M2 emulsion. After storage for 28 days, there was a great difference. The EI value of M1 emulsion increased significantly, and the network structure increased significantly. The EI value of M2 emulsion changed little during the monitoring time. The results could be predicted that gelation would occur earlier in M1 emulsion, and M2 emulsion would retain its original fluidity in a long time.

**TABLE 2:** Elasticity index of M1 emulsion and M2 emulsion under different storage time.

Emulsion	SA concentration (%)	EI (×10^–3^ nm^–2^)				
		D0	D14	D28	D42	D56
M1	0.75	3.19 ± 0.43	6.27 ± 0.62	23.01 ± 1.18	29.99 ± 2.80	33.72 ± 0.74
	1	5.97 ± 0.64	6.51 ± 0.40	23.96 ± 1.76	27.92 ± 1.29	33.40 ± 0.98
	1.25	7.47 ± 0.74	12.65 ± 1.01	33.91 ± 2.37	33.36 ± 0.18	37.94 ± 0.25
	1.5	8.16 ± 0.79	10.44 ± 0.61	22.92 ± 1.69	29.55 ± 2.17	33.23 ± 3.10
M2	0.75	2.53 ± 0.07	3.61 ± 0.06	4.17 ± 0.12	4.48 ± 0.20	5.34 ± 0.23
	1	2.61 ± 0.23	3.72 ± 0.08	4.69 ± 0.21	4.95 ± 0.13	5.66 ± 0.19
	1.25	3.43 ± 0.09	5.35 ± 0.09	5.69 ± 0.50	7.43 ± 0.13	7.50 ± 0.17
	1.5	3.93 ± 0.18	4.79 ± 0.18	4.81 ± 0.39	7.12 ± 0.16	8.37 ± 0.18

D0, D14, D28, D42, and D56 represent different storage times of emulsions.

SA represents sodium alginate and EI represents the elasticity index.

As shown in Table 3, the MVI value was similar to the change trend of EI value. The viscosity of M1 emulsion and M2 emulsion increases in different degrees during storage, indicating that the emulsion structure was constantly changing during this process. When storage time was extended from 0 to 56 days, the MVI value of M1 emulsion added 0.75% alginate was 0.04 × 10^–2^ nm^–2^ s increased to 157.78 × 10^–2^ nm^–2^ s, so its viscosity increased with the increase of storage time. The increase in MVI value was due to the gradual formation of the dense gel network in the emulsion structure, which brought higher movement resistance between droplets. It was manifested as an increase in viscosity and elasticity in a macroscopic view. In addition, comparing the MVI value of M1 and M2 emulsions, it was found that M1 emulsion increased significantly during the monitoring period, while the MVI value of M2 emulsion changed relatively little. It showed that the viscosity of M1 emulsion increased with the increase of storage time, and M2 emulsion tended to maintain its original viscosity during storage.

**TABLE 3:** Macroscopic viscosity index of M1 and M2 emulsion under different storage time.

Emulsion	SA concentration (%)	MVI (×10^–2^ nm^–2^ s)				
		D0	D14	D28	D42	D56
M1	0.75	0.04 ± 0.02	0.15 ± 0.03	4.06 ± 2.58	6.97 ± 2.82	157.78 ± 40.97
	1	0.33 ± 0.06	0.18 ± 0.05	3.86 ± 1.02	7.07 ± 3.72	48.27 ± 26.98
	1.25	0.35 ± 0.06	0.34 ± 0.03	5.44 ± 1.09	11.27 ± 3.74	60.02 ± 48.17
	1.5	0.53 ± 0.09	0.43 ± 0.05	3.02 ± 0.26	17.52 ± 3.50	48.27 ± 26.98
M2	0.75	0.05 ± 0.01	1.40 ± 0.26	0.46 ± 0.09	4.33 ± 1.42	29.25 ± 2.32
	1	0.16 ± 0.05	0.50 ± 0.05	0.88 ± 0.15	1.84 ± 0.24	7.78 ± 1.51
	1.25	0.34 ± 0.05	0.29 ± 0.03	0.47 ± 0.12	3.33 ± 0.27	4.10 ± 0.26
	1.5	0.33 ± 0.06	0.46 ± 0.04	0.46 ± 0.09	2.27 ± 0.13	6.73 ± 0.44

D0, D14, D28, D42, and D56 represent different storage times of emulsions.

SA represents sodium alginate and MVI represents the macroscopic viscosity index.

To sum up, the preparation methods of emulsion had an important effect on the rheological properties of emulsion. It showed that M1 emulsion could reach solid-liquid equilibrium earlier, and had stronger viscoelasticity, while M2 emulsion had better fluidity.

### Interaction of calcium carbonate and sodium alginate in aqueous phase

The different behavior of M1 and M2 emulsions might be related to the interaction between SA and CaCO_3_ in water phase. In order to further analyze the reasons for the good storage stability of M2 emulsion, TSI and Zeta potential of CaCO_3_/SA in aqueous phase were measured and the microstructure of that were observed.

**FIGURE 8:**
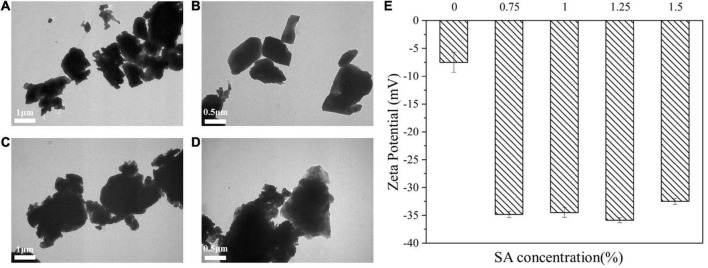
TEM images of CaCO_3_ suspension **(A,B)** and CaCO_3_/SA suspension **(C,D)**. Zeta potential **(E)** of CaCO_3_ suspension and CaCO_3_/SA suspension. The measurement results were the average values of triplicate measurements.

It could be seen from Figures 8A,B that CaCO_3_ particle without SA was a cube. Figures 8C,D showed the microscopic morphology of CaCO_3_ particle after the addition of SA, and it could be observed that SA uniformly covered the surface of CaCO_3_. It illustrated that CaCO_3_ had sufficient contact with SA.

As shown in Figure 8E, the addition of SA could significantly improve the absolute value of Zeta potential. The Zeta potential reflects the stability of the dispersion system, that is, the stability of the dispersion system reduces with the decrease of the absolute value of Zeta potential (35). It confirmed that the addition of SA enhanced the stability of CaCO_3_ suspension. It might be due to the electrostatic repulsion of CaCO_3_/SA suspension was enhanced, which prevented the agglomeration and settlement of particles and facilitated the dispersion of CaCO_3_ particles in water phase. As previously described, the improved anti-aggregation stability of CaCO_3_/SA suspension and M2 emulsion could be attributed to the interaction between CaCO_3_ and SA.

**FIGURE 9:**
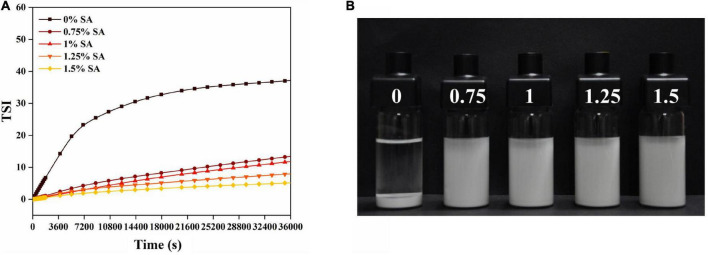
Turbiscan stability index **(A)** and photograph after 10 h of storage **(B)** of CaCO_3_/SA suspensions of different SA concentrations.

**FIGURE 10:**
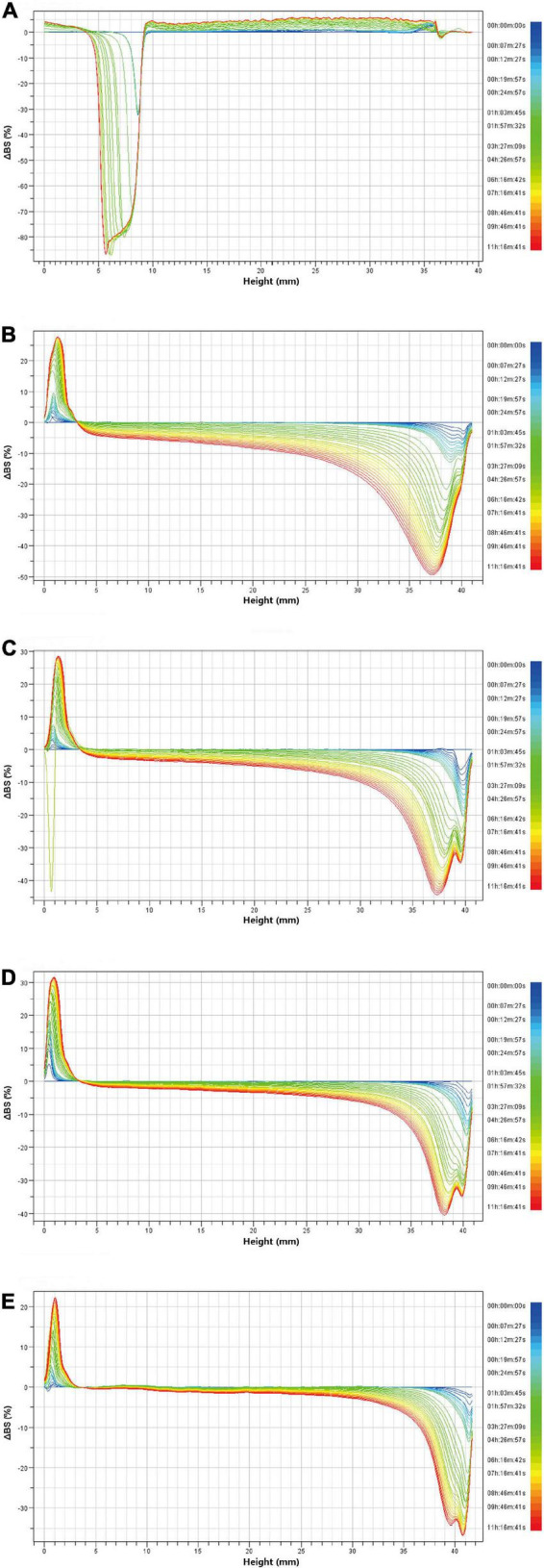
Delta backscattering profiles of CaCO_3_/SA suspensions with different SA concentrations: the suspension without SA **(A)**; the suspension with 0.75% SA **(B)**, 1% SA **(C)**, 1.25% SA **(D)**, and 1.5% SA **(E)**.

Turbiscan stability index is also an important parameter to characterize the stability of CaCO_3_ suspension. Figure 9A showed the change of TSI index within 10 h after suspension preparation. The TSI value of CaCO_3_ suspension without SA increased rapidly in a short time and was significantly higher than that of CaCO_3_ suspension with SA. As shown in Figure 9B, the CaCO_3_ suspension without SA was completely stratified after storage for 10 h, and CaCO_3_ particles were deposited at the bottom. The CaCO_3_ suspension with SA still showed good dispersibility. These results indicated that the CaCO_3_ suspension without SA was very unstable. In addition, the TSI value decreased with the increase of SA concentration. Especially when SA concentration was 1.5%, the TSI value was close to zero (Figure 9A). From a macro perspective, CaCO_3_ particles tended to be suspended in the aqueous phase rather than deposited at the bottom with the increase of SA concentration (Figure 9B). Therefore, the addition of SA was beneficial to improve the stability of CaCO_3_ suspension, and the increase of SA concentration further deepened this stability. Figure 10 showed the delta backscattering profiles of CaCO_3_/SA suspensions with different SA concentrations. It can be seen that Δ*BS* of the CaCO_3_/SA suspensions decreased with the increase of SA concentration in the measurement cell height range of 0 mm to 3 mm. In the middle of the measuring cell, Δ*BS* tended to 0 with the increase of SA concentration. In the upper of the measuring cell, Δ*BS* showed a downward trend. These phenomena indicated that the precipitation of the suspension was reduced and had better dispersion. It is also confirmed that the addition of SA was beneficial to improve the stability of CaCO_3_/SA suspensions. The reason might be due to the addition of SA decreased the surface energy of CaCO_3_, which was more conducive to its dispersion in aqueous phase. Moreover, SA increased the viscosity of aqueous solution and limited the aggregation and settlement of CaCO_3_.

## Conclusion

This paper studied the effects of two-step emulsifying method and one-step emulsifying method on the physicochemical properties of M1 emulsion and M2 emulsion stabilized by CaCO_3_ and SA. Our results demonstrated that the droplet size of M1 emulsion was 21.78–49.62 μm, the droplet size of M2 emulsion was 6.50–11.87 μm. Additionally, the droplet size of the emulsion decreased with the increase of SA concentration. By observing the microstructure of the emulsion, it is clear that M1 emulsion and M2 emulsion always maintain their original microstructure during storage from 0 to 28 days. The rheological and microrheological properties of the emulsion showed that M1 emulsion reached the solid-liquid equilibrium earlier and had stronger viscoelasticity. In addition, the network structure would gradually form during storage, which made the emulsion gradually show a gel-like behavior. However, M2 emulsion could maintain good fluidity in storage, which was related to the interaction between SA and CaCO_3_ in the aqueous phase. To sum up, Pickering emulsion with better stability can be achieved in a simple way by adjusting the method of making emulsion and the order of adding materials. Different emulsification methods have a certain impact on the physicochemical properties and stability of the emulsion. It provides a new idea for regulating the physicochemical properties and storage properties of Pickering emulsion in the future.

## Data availability statement

The raw data supporting the conclusions of this article will be made available by the authors, without undue reservation.

## Author contributions

XTY: methodology, data curation, formal analysis, investigation, and writing—original draft and review and editing. HS: methodology, data curation, formal analysis, and investigation. HL and XXY: methodology, formal analysis, and writing—review and editing. BL: methodology and data curation. JL: conceptualization, methodology, data curation, investigation, writing—original draft and review and editing, supervision, and funding acquisition. All authors contributed to the article and approved the submitted version.
